# Exponential Entropy for Simplified Neutrosophic Sets and Its Application in Decision Making

**DOI:** 10.3390/e20050357

**Published:** 2018-05-10

**Authors:** Jun Ye, Wenhua Cui

**Affiliations:** Department of Electrical and Information Engineering, Shaoxing University, Shaoxing 312000, China

**Keywords:** simplified neutrosophic exponential entropy, simplified neutrosophic set, single-valued neutrosophic set, interval-valued neutrosophic set, decision making

## Abstract

Entropy is one of many important mathematical tools for measuring uncertain/fuzzy information. As a subclass of neutrosophic sets (NSs), simplified NSs (including single-valued and interval-valued NSs) can describe incomplete, indeterminate, and inconsistent information. Based on the concept of fuzzy exponential entropy for fuzzy sets, this work proposes exponential entropy measures of simplified NSs (named simplified neutrosophic exponential entropy (SNEE) measures), including single-valued and interval-valued neutrosophic exponential entropy measures, and investigates their properties. Then, the proposed exponential entropy measures of simplified NSs are compared with existing related entropy measures of interval-valued NSs to illustrate the rationality and effectiveness of the proposed SNEE measures through a numerical example. Finally, the developed exponential entropy measures for simplified NSs are applied to a multi-attribute decision-making example in an interval-valued NS setting to demonstrate the application of the proposed SNEE measures. However, the SNEE measures not only enrich the theory of simplified neutrosophic entropy, but also provide a novel way of measuring uncertain information in a simplified NS setting.

## 1. Introduction

Entropy is an important mathematical tool for measuring the fuzziness of a fuzzy event. Zadeh [1] first presented the entropy of a fuzzy event, based on a probabilistic framework, in 1968 to measure the fuzziness of a fuzzy event. Then, De Luca and Termini [2] defined the entropy of a fuzzy set (FS) based on Shannon’s function, and formulated the axioms of a fuzzy entropy measure. Pal and Pal [3] proposed fuzzy exponential entropy measures for fuzzy sets. Later, Verma and Sharma [4] presented a generalized exponential fuzzy entropy measure. Since intuitionistic FSs [5] and interval-valued intuitionistic FSs [6] are extension forms of FSs, Bustince and Burrillo [7] introduced intuitionistic fuzzy and interval-valued fuzzy entropy measures as an extension of fuzzy entropy. Szmidt and Kacprzyk [8] proposed intuitionistic fuzzy entropy based on an extension of De Luca and Termini’s [2] axioms of fuzzy entropy. Subsequently, Vlachos and Sergiagis [9] presented an intuitionistic fuzzy entropy measure and indicated an intuitive and mathematical connection between the notions of fuzzy entropy and intuitionistic fuzzy entropy. Zhang and Jiang [10] put forward an entropy measure of vague sets (intuitionistic FSs) as an extension of logarithmic fuzzy entropy [2]. Furthermore, Ye [11] proposed cosine and sine entropy measures of intuitionistic FSs. As a generalization of fuzzy exponential entropy in Reference [3], Verma and Sharma [12] introduced an intuitionistic fuzzy exponential entropy measure based on the concept of fuzzy entropy. Thereafter, Verma and Sharma [13,14] introduced intuitionistic fuzzy entropy measures of order-alpha and R-norm. Ye [15] presented an entropy measure of interval-valued intuitionistic FSs. Wei et al. [16] defined a similarity measure and entropy of interval-valued intuitionistic FSs, and revealed their relationship. Zhang et al. [17] defined an axiom of interval-valued intuitionistic fuzzy distance-based entropy and some entropy measures for interval-valued intuitionistic FSs, along with their relationship, based on inclusion measures, and similarity measures of interval-valued intuitionistic FSs.

Since there is incomplete, indeterminate, and inconsistent information in the real world, Smaradache [18] presented the concept of the neutrosophic set (NS), which is described independently by truth, falsity, and indeterminacy membership functions defined in real standard [0, 1] or non-standard ]^−^0, 1^+^[ intervals. For convenient engineering applications of NSs, some researchers constrained the membership functions of truth, falsity, and indeterminacy in a real standard interval [0, 1], and defined single-valued NSs [19], interval-valued NSs [20], and simplified NSs (containing single-valued and interval-valued NSs) [21] as subclasses of NSs, which generalize intuitionistic FSs and interval-valued intuitionistic FSs. As a generalization of intuitionistic fuzzy entropy and similarity measures, Majumder and Samanta [22] developed some similarity measures and an entropy measure for single-valued NSs. Aydoğdu [23] then proposed entropy and similarity measures of interval-valued NSs, and indicated their relationship. Ye and Du [24] defined distances, similarity measures, and entropy for interval-valued NSs, and indicated their relationship.

However, there is no study on exponential entropy measures for simplified NSs (single-valued and interval-valued NSs) in existing literature. Motivated by exponential entropy measures of FSs, this paper attempts to present exponential entropy measures of simplified NSs (named simplified neutrosophic exponential entropy (SNEE) measures), including single-valued and interval-valued neutrosophic exponential entropy measures and their application in decision making. In order to do so, the paper is organized as follows: Section 2 briefly describes some basic concepts related to simplified NS theory. In Section 3, SNEE measures are proposed, including single-valued neutrosophic exponential entropy and interval-valued neutrosophic exponential entropy measures, which satisfy the axiomatic requirements [24], and then, some mathematical properties of the proposed exponential entropy measures are investigated. Section 4 gives a comparison of various entropy measures of interval-valued NSs introduced in References [23,24] through a numerical example, to show the effectiveness of the proposed exponential entropy. In Section 5, the proposed SNEE measures are applied to an actual example of decision making in an interval-valued NS environment. Section 6 contains the conclusions of this study and future work.

## 2. Preliminaries of Simplified Neutrosophic Sets

In 2014, Ye [21] presented the concept of simplified NSs, including single-valued and interval-valued NSs, as a subclass of NSs, in order to be conveniently applied in science and engineering fields. A simplified NS, *S*, is defined as *S* = {<*x_j_*, *T_S_*(*x_j_*), *I_S_*(*x_j_*), *F_S_*(*x_j_*)>| *x_j_* ∊ *X*} in a universal set, *X* = {*x*_1_, *x*_2_, …, *x_n_*}, which is described independently by *T_S_*(*x_j_*), *I_S_*(*x_j_*), *F_S_*(*x_j_*) ∊ [0, 1] for a single-valued NS and *T_S_*(*x_j_*) = $\left[T_{S}^{L}(x_{j}),T_{S}^{U}(x_{j})\right]$, *I_S_*(*x_j_*) = $\left[I_{S}^{L}(x_{j}),I_{S}^{U}(x_{j})\right]$, *F_S_*(*x_j_*) = $\left[F_{S}^{L}(x_{j}),F_{S}^{U}(x_{j})\right]$ ⊆ [0, 1] for an interval-valued NS, satisfying the conditions $0\leq T_{S}^{}(x_{j})+I_{S}^{}(x_{j})+F_{S}^{}(x_{j})\leq 1$ for a single-valued NS and $0\leq T_{S}^{U}(x_{j})+I_{S}^{U}(x_{j})+F_{S}^{U}(x_{j})\leq 1$ for an interval-valued NS. 

Let SNS(*X*) denote the family of all simplified NSs in the universe set, *X* = {*x*_1_, *x*_2_, …, *x_n_*}, and set *N*, *S* ∊ SNS(*X*) as *N* = {<*x_j_*, *T_N_*(*x_j_*), *I_N_*(*x_j_*), *F_N_*(*x_j_*)>| *x_j_* ∊ *X*} and *S* = {<*x_j_*, *T_S_*(*x_j_*), *I_S_*(*x_j_*), *F_S_*(*x_j_*)>| *x_j_* ∊ *X*}. Then, some operations of simplified NSs can be defined as follows [21,25]:

1. *N* ⊆ *S* if and only if *T_N_*(*x_j_*) ≤ *T_S_*(*x_j_*), *I_N_*(*x_j_*) ≥ *I_S_*(*x_j_*), and *F_N_*(*x_j_*) ≥ *F_S_*(*x_j_*) for single-valued NSs, and $T_{N}^{L}(x_{j})\leq T_{S}^{L}(x_{j})$, $T_{N}^{U}(x_{j})\leq T_{S}^{U}(x_{j})$, $I_{N}^{L}(x_{j})\geq I_{S}^{L}(x_{j})$, $I_{N}^{U}(x_{j})\geq I_{S}^{U}(x_{j})$, $F_{N}^{L}(x_{j})\geq F_{S}^{L}(x_{j})$, and $F_{N}^{U}(x_{j})\geq F_{S}^{U}(x_{j})$ for interval-valued NSs, and *x_j_* ∊ *X*;
2. *N* = *S* if and only if *N* ⊆ *S* and *S* ⊆ *N*;
3. *S^c^* = {<*x_j_*, *F_S_*(*x_j_*), 1 − *I_S_*(*x_j_*), *T_S_*(*x_j_*)>| *x_j_* ∊ *X*} for the complement of the single-valued NS, *S*, and $S^{c}=\{<x_{j},[F_{S}^{L}(x_{j}),F_{S}^{U}(x_{j})],[1-I_{S}^{U}(x_{j}),1-I_{S}^{L}(x_{j})],[T_{S}^{L}(x_{j}),T_{S}^{U}(x_{j})]>|x_{j}\in X\}$ for the complement of the interval-valued NS, *S*;
4. $N\cup S=\{<x_{j},T_{N}^{}(x_{j})\vee T_{S}^{}(x_{j}),I_{N}^{}(x_{j})\wedge I_{S}^{}(x_{j}),F_{N}^{}(x_{j})\wedge F_{S}^{}(x_{j})>|x_{j}\in X\}$ for single-valued NSs, and $N\cup S=\left\{\begin{array}{l} <x_{j},[T_{N}^{L}(x_{j})\vee T_{S}^{L}(x_{j}),T_{N}^{U}(x_{j})\vee T_{S}^{U}(x_{j})],\\ {}[I_{N}^{L}(x_{j})\wedge I_{S}^{L}(x_{j}),I_{N}^{U}(x_{j})\wedge I_{S}^{U}(x_{j})],\\ {}[F_{N}^{L}(x_{j})\wedge F_{S}^{L}(x_{j}),F_{N}^{U}(x_{j})\wedge F_{S}^{U}(x_{j})]>|x_{j}\in X \end{array}\right\}$ for interval-valued NSs;
5. $N\cap S=\{<x_{j},T_{N}^{}(x_{j})\wedge T_{S}^{}(x_{j}),I_{N}^{}(x_{j})\vee I_{S}^{}(x_{j}),F_{N}^{}(x_{j})\vee F_{S}^{}(x_{j})>|x_{j}\in X\}$ for single-valued NSs, and $N\cap S=\left\{\begin{array}{l} <x_{j},[T_{N}^{L}(x_{j})\wedge T_{S}^{L}(x_{j}),T_{N}^{U}(x_{j})\wedge T_{S}^{U}(x_{j})],\\ {}[I_{N}^{L}(x_{j})\vee I_{S}^{L}(x_{j}),I_{N}^{U}(x_{j})\vee I_{S}^{U}(x_{j})],\\ {}[F_{N}^{L}(x_{j})\vee F_{S}^{L}(x_{j}),F_{N}^{U}(x_{j})\vee F_{S}^{U}(x_{j})]>|x_{j}\in X \end{array}\right\}$ for interval-valued NSs;
6. $N⊕S=\left\{\langle x_{j},T_{N}^{}(x_{j})+T_{S}^{}(x_{j})-T_{N}^{}(x_{j})T_{S}^{}(x_{j}),I_{N}^{}(x_{j})I_{S}^{}(x_{j}),F_{N}^{}(x_{j})F_{S}^{}(x_{j})\rangle |x_{j}\in X\right\}$ for single-valued NSs, and $N⊕S=\left\{\langle \begin{array}{l} x_{j},[T_{N}^{L}(x_{j})+T_{S}^{L}(x_{j})-T_{N}^{L}(x_{j})T_{S}^{L}(x_{j}),T_{N}^{U}(x_{j})+T_{S}^{U}(x_{j})-T_{N}^{U}(x_{j})T_{S}^{U}(x_{j})],\\ \left[I_{N}^{L}(x_{j})I_{S}^{L}(x_{j}),I_{N}^{U}(x_{j})I_{S}^{U}(x_{j})],[F_{N}^{L}(x_{j})F_{S}^{L}(x_{j}),F_{N}^{U}(x_{j})F_{S}^{U}(x_{j})\right] \end{array}\rangle |x_{j}\in X\right\}$ for interval-valued NSs;
7. $N⊗S=\left\{\langle \begin{array}{l} x_{j},T_{N}^{}(x_{j})T_{S}^{}(x_{j}),I_{N}^{}(x_{j})+I_{S}^{}(x_{j})-I_{N}^{}(x_{j})I_{S}^{}(x_{j}),\\ F_{N}^{}(x_{j})+F_{S}^{}(x_{j})-F_{N}^{}(x_{j})F_{S}^{}(x_{j}), \end{array}\rangle |x_{j}\in X\right\}$ for single-valued NSs, and $N⊗S=\left\{\langle \begin{array}{l} x_{j},[T_{N}^{L}(x_{j})T_{S}^{L}(x_{j}),T_{N}^{U}(x_{j})T_{N}^{U}(x_{j})],[I_{N}^{L}(x_{j})+I_{S}^{L}(x_{j})-I_{N}^{L}(x_{j})I_{S}^{L}(x_{j}),\\ I_{N}^{U}(x_{j})+I_{S}^{U}(x_{j})-I_{N}^{U}(x_{j})I_{S}^{U}(x_{j})],[F_{N}^{L}(x_{j})+F_{S}^{L}(x_{j})-F_{N}^{L}(x_{j})F_{S}^{L}(x_{j}),\\ F_{N}^{U}(x_{j})+F_{S}^{U}(x_{j})-F_{N}^{U}(x_{j})F_{S}^{U}(x_{j})] \end{array}\rangle |x_{j}\in X\right\}$ for interval-valued NSs;
8. $\delta S=\left\{\langle x_{j},1-{\left(1-T_{S}^{}(x_{j})\right)}^{\delta },I_{S}^{\delta }(x_{j}),F_{S}^{\delta }(x_{j})\rangle |x_{j}\in X\right\}$ for the single-valued NS, *S*, and *δ* > 0, and $\delta S=\left\{\langle \begin{array}{l} x_{j},[1-{\left(1-T_{S}^{L}(x_{j})\right)}^{\delta },1-{\left(1-T_{S}^{U}(x_{j})\right)}^{\delta }],\\ \left[{\left(I_{S}^{L}(x_{j})\right)}^{\delta },{\left(I_{S}^{U}(x_{j})\right)}^{\delta }],[{\left(F_{S}^{L}(x_{j})\right)}^{\delta },{\left(F_{S}^{U}(x_{j})\right)}^{\delta }\right] \end{array}\rangle |x_{j}\in X\right\}$ for the interval-valued NS, *S*, and *δ* > 0;
9. $S^{\delta }=\left\{\langle x_{j},T_{S}^{\delta }(x_{j}),1-{\left(1-I_{S}^{}(x_{j})\right)}^{\delta },1-{\left(1-F_{S}^{}(x_{j})\right)}^{\delta }\rangle |x_{j}\in X\right\}$ for the single-valued NS, *S*, and *δ* > 0, and $S^{\delta }=\left\{\langle \begin{array}{l} x_{j},[{\left(T_{S}^{L}(x_{j})\right)}^{\delta },{\left(T_{S}^{U}(x_{j})\right)}^{\delta }],[1-{\left(1-I_{S}^{L}(x_{j})\right)}^{\delta },1-{\left(1-I_{S}^{U}(x_{j})\right)}^{\delta }],\\ \left[1-{\left(1-F_{S}^{L}(x_{j})\right)}^{\delta },1-{\left(1-F_{S}^{U}(x_{j})\right)}^{\delta }\right] \end{array}\rangle |x_{j}\in X\right\}$ for the interval-valued NS, *S*, and *δ* > 0.

## 3. Simplified Neutrosophic Exponential Entropy

For a fuzzy set, *A* = {*x_j_*, *T_A_*(*x_j_*)| *x_j_* ∊ *X*}, in a universal set, *X* = {*x*_1_, *x*_2_, …, *x_n_*}, Pal and Pal [3] introduced fuzzy exponential entropy for *A*:

$$H(A)=\frac{1}{n\left(\sqrt{e}-1\right)}\sum_{j=1}^{n}\left[T_{A}\left(x_{j}\right)e^{\left(1-T_{A}\left(x_{j}\right)\right)}+\left(1-T_{A}\left(x_{j}\right)\right)e^{T_{A}\left(x_{j}\right)}-1\right].$$

Based on the extension of fuzzy exponential entropy, we can propose SNEE for a simplified NS.

**Definition** **1.** *Let S = {<x_j_, T_S_(x_j_), I_S_(x_j_), F_S_(x_j_)>| x_j_* *∊ X} be a simplified NS in a universal set X = {x_1_, x_2_,…, x_n_}. Then, the SNEE measure of S is defined as the following two forms:*

(1) $$Y_{1}(S)=\frac{1}{3n\left(\sqrt{e}-1\right)}\sum_{j=1}^{n}\left[\begin{array}{l} T_{S}\left(x_{j}\right)e^{\left(1-T_{S}\left(x_{j}\right)\right)}+\left(1-T_{S}\left(x_{j}\right)\right)e^{T_{S}\left(x_{j}\right)}-1\\ +I_{S}\left(x_{j}\right)e^{\left(1-I_{S}\left(x_{j}\right)\right)}+\left(1-I_{S}\left(x_{j}\right)\right)e^{I_{S}\left(x_{j}\right)}-1\\ +F_{S}\left(x_{j}\right)e^{\left(1-F_{S}\left(x_{j}\right)\right)}+\left(1-F_{S}\left(x_{j}\right)\right)e^{F_{S}\left(x_{j}\right)}-1 \end{array}\right]\;\mathrm{for}\;\mathrm{the}\;\mathrm{single}-\mathrm{valued}\;\mathrm{NS},\;S,$$

(2) $$Y_{2}(S)=\frac{1}{6n\left(\sqrt{e}-1\right)}\sum_{j=1}^{n}\left[\begin{array}{l} T_{S}^{L}\left(x_{j}\right)e^{\left(1-T_{S}^{L}\left(x_{j}\right)\right)}+\left(1-T_{S}^{L}\left(x_{j}\right)\right)e^{T_{S}^{L}\left(x_{j}\right)}-1\\ +I_{S}^{L}\left(x_{j}\right)e^{\left(1-I_{S}^{L}\left(x_{j}\right)\right)}+\left(1-I_{S}^{L}\left(x_{j}\right)\right)e^{I_{S}^{L}\left(x_{j}\right)}-1\\ +F_{S}^{L}\left(x_{j}\right)e^{\left(1-F_{S}^{L}\left(x_{j}\right)\right)}+\left(1-F_{S}^{L}\left(x_{j}\right)\right)e^{F_{S}^{L}\left(x_{j}\right)}-1\\ +T_{S}^{U}\left(x_{j}\right)e^{\left(1-T_{S}^{U}\left(x_{j}\right)\right)}+\left(1-T_{S}^{U}\left(x_{j}\right)\right)e^{T_{S}^{U}\left(x_{j}\right)}-1\\ +I_{S}^{U}\left(x_{j}\right)e^{\left(1-I_{S}^{U}\left(x_{j}\right)\right)}+\left(1-I_{S}^{U}\left(x_{j}\right)\right)e^{I_{S}^{U}\left(x_{j}\right)}-1\\ +F_{S}^{U}\left(x_{j}\right)e^{\left(1-F_{S}^{U}\left(x_{j}\right)\right)}+\left(1-F_{S}^{U}\left(x_{j}\right)\right)e^{F_{S}^{U}\left(x_{j}\right)}-1 \end{array}\right]\;\mathrm{for}\;\mathrm{the}\;\mathrm{interval}-\mathrm{valued}\;\mathrm{NS},\;S.$$

Corresponding to an axiomatic definition of an entropy measure for interval-valued NSs introduced by Ye and Du [3], the exponential entropy measure of a simplified NS can be given by the following theorem.

**Theorem** **1.** *For any S* *∊ SNS(X), Y_k_(S) for k = 1, 2 is the SNEE measure of the simplified NS, S, which satisfies the following properties, (P1)–(P4):*

*(P1)*  Y_k_(S) = 0 if S is a crisp set;
*(P2)*  Y_k_(S) = 1 if and only if S = A = {<x_j_, [0.5, 0.5], [0.5, 0.5], [0.5, 0.5]>|x_j_ ∊ X} for interval-valued NSs, or S = A = {<x_j_, 0.5, 0.5, 0.5>|x_j_ ∊ X} for single-valued NSs;
*(P3)*  If the closer a simplified NS, S, is to A than P, the fuzzier S is than P, then Y_k_(P) ≤ Y_k_(S) for P ∊ SNS(X);
*(P4)*  Y_k_(S) = Y_k_(S^c^) if S^c^ is the complement of S.

**Proof.**  (P1) If *S* is a crisp set, i.e., *S* = {<*x_j_*, 1, 0, 0>|*x_j_* ∊ *X*} or *S* = {<*x_j_*, 0, 0, 1>|*x_j_* ∊ *X*} for a single-valued NS, *S*, ∊ SNS(*X*) and *x_j_* ∊ *X,* and *S* = {<*x_j_*, [1, 1], [0, 0], [0, 0]>|*x_j_* ∊ *X*} or *S* = {<*x_j_*, [0, 0], [0, 0], [1, 1]>|*x_j_* ∊ *X*} for an interval-valued NS, *S*, ∊ SNS(*X*) and *x_j_* ∊ *X*, by using Equation (1) for *S* = {<*x_j_*, 1, 0, 0>|*x_j_* ∊ *X*} or *S* = {<*x_j_*, 0, 0, 1>|*x_j_* ∊ *X*}, we have the following results:

$$Y_{1}(S)=\frac{1}{3n\left(\sqrt{e}-1\right)}\sum_{j=1}^{n}\left[\begin{array}{l} T_{S}\left(x_{j}\right)e^{\left(1-T_{S}\left(x_{j}\right)\right)}+\left(1-T_{S}\left(x_{j}\right)\right)e^{T_{S}\left(x_{j}\right)}-1\\ +I_{S}\left(x_{j}\right)e^{\left(1-I_{S}\left(x_{j}\right)\right)}+\left(1-I_{S}\left(x_{j}\right)\right)e^{I_{S}\left(x_{j}\right)}-1\\ +F_{S}\left(x_{j}\right)e^{\left(1-F_{S}\left(x_{j}\right)\right)}+\left(1-F_{S}\left(x_{j}\right)\right)e^{F_{S}\left(x_{j}\right)}-1 \end{array}\right]=\frac{1}{3n\left(\sqrt{e}-1\right)}\sum_{j=1}^{n}\left[\begin{array}{l} e^{\left(1-1\right)}+\left(1-1\right)e^{}-1\\ +0e^{}+\left(1-0\right)e^{0}-1\\ +0e^{\left(1-0\right)}+\left(1-0\right)e^{0}-1 \end{array}\right]=0$$

or

$$Y_{1}(S)=\frac{1}{3n\left(\sqrt{e}-1\right)}\sum_{j=1}^{n}\left[\begin{array}{l} T_{S}\left(x_{j}\right)e^{\left(1-T_{S}\left(x_{j}\right)\right)}+\left(1-T_{S}\left(x_{j}\right)\right)e^{T_{S}\left(x_{j}\right)}-1\\ +I_{S}\left(x_{j}\right)e^{\left(1-I_{S}\left(x_{j}\right)\right)}+\left(1-I_{S}\left(x_{j}\right)\right)e^{I_{S}\left(x_{j}\right)}-1\\ +F_{S}\left(x_{j}\right)e^{\left(1-F_{S}\left(x_{j}\right)\right)}+\left(1-F_{S}\left(x_{j}\right)\right)e^{F_{S}\left(x_{j}\right)}-1 \end{array}\right]=\frac{1}{3n\left(\sqrt{e}-1\right)}\sum_{j=1}^{n}\left[\begin{array}{l} 0e^{\left(1-0\right)}+\left(1-0\right)e^{0}-1\\ +0e^{}+\left(1-0\right)e^{0}-1\\ +e^{\left(1-1\right)}+\left(1-1\right)e-1 \end{array}\right]=0,$$

and then by using Equation (2) for *S* = {<*x_j_*, [1, 1], [0, 0], [0, 0]>|*x_j_* ∊ *X*} or *S* = {<*x_j_*, [0, 0], [0, 0], [1, 1]>|*x_j_* ∊ *X*}, we have the following results:

$$Y_{2}(S)=\frac{1}{6n\left(\sqrt{e}-1\right)}\sum_{j=1}^{n}\left[\begin{array}{l} T_{S}^{L}\left(x_{j}\right)e^{\left(1-T_{S}^{L}\left(x_{j}\right)\right)}+\left(1-T_{S}^{L}\left(x_{j}\right)\right)e^{T_{S}^{L}\left(x_{j}\right)}-1\\ +I_{S}^{L}\left(x_{j}\right)e^{\left(1-I_{S}^{L}\left(x_{j}\right)\right)}+\left(1-I_{S}^{L}\left(x_{j}\right)\right)e^{I_{S}^{L}\left(x_{j}\right)}-1\\ +F_{S}^{L}\left(x_{j}\right)e^{\left(1-F_{S}^{L}\left(x_{j}\right)\right)}+\left(1-F_{S}^{L}\left(x_{j}\right)\right)e^{F_{S}^{L}\left(x_{j}\right)}-1\\ +F_{S}^{L}\left(x_{j}\right)e^{\left(1-F_{S}^{L}\left(x_{j}\right)\right)}+\left(1-F_{S}^{L}\left(x_{j}\right)\right)e^{F_{S}^{L}\left(x_{j}\right)}-1\\ +T_{S}^{U}\left(x_{j}\right)e^{\left(1-T_{S}^{U}\left(x_{j}\right)\right)}+\left(1-T_{S}^{U}\left(x_{j}\right)\right)e^{T_{S}^{U}\left(x_{j}\right)}-1\\ +I_{S}^{U}\left(x_{j}\right)e^{\left(1-I_{S}^{U}\left(x_{j}\right)\right)}+\left(1-I_{S}^{U}\left(x_{j}\right)\right)e^{I_{S}^{U}\left(x_{j}\right)}-1\\ +F_{S}^{U}\left(x_{j}\right)e^{\left(1-F_{S}^{U}\left(x_{j}\right)\right)}+\left(1-F_{S}^{U}\left(x_{j}\right)\right)e^{F_{S}^{U}\left(x_{j}\right)}-1 \end{array}\right]=\frac{1}{6n\left(\sqrt{e}-1\right)}\sum_{j=1}^{n}\left[\begin{array}{l} e^{\left(1-1\right)}+\left(1-1\right)e^{}-1\\ +0e^{\left(1-0\right)}+\left(1-0\right)e^{0}-1\\ +0e^{\left(1-0\right)}+\left(1-0\right)e^{0}-1\\ +e^{\left(1-1\right)}+\left(1-1\right)e-1\\ +0e^{\left(1-0\right)}+\left(1-0\right)e^{0}-1\\ +0e^{\left(1-0\right)}+\left(1-0\right)e^{0}-1 \end{array}\right]=0$$

or

$$Y_{2}(S)=\frac{1}{6n\left(\sqrt{e}-1\right)}\sum_{j=1}^{n}\left[\begin{array}{l} T_{S}^{L}\left(x_{j}\right)e^{\left(1-T_{S}^{L}\left(x_{j}\right)\right)}+\left(1-T_{S}^{L}\left(x_{j}\right)\right)e^{T_{S}^{L}\left(x_{j}\right)}-1\\ +I_{S}^{L}\left(x_{j}\right)e^{\left(1-I_{S}^{L}\left(x_{j}\right)\right)}+\left(1-I_{S}^{L}\left(x_{j}\right)\right)e^{I_{S}^{L}\left(x_{j}\right)}-1\\ +F_{S}^{L}\left(x_{j}\right)e^{\left(1-F_{S}^{L}\left(x_{j}\right)\right)}+\left(1-F_{S}^{L}\left(x_{j}\right)\right)e^{F_{S}^{L}\left(x_{j}\right)}-1\\ +F_{S}^{L}\left(x_{j}\right)e^{\left(1-F_{S}^{L}\left(x_{j}\right)\right)}+\left(1-F_{S}^{L}\left(x_{j}\right)\right)e^{F_{S}^{L}\left(x_{j}\right)}-1\\ +T_{S}^{U}\left(x_{j}\right)e^{\left(1-T_{S}^{U}\left(x_{j}\right)\right)}+\left(1-T_{S}^{U}\left(x_{j}\right)\right)e^{T_{S}^{U}\left(x_{j}\right)}-1\\ +I_{S}^{U}\left(x_{j}\right)e^{\left(1-I_{S}^{U}\left(x_{j}\right)\right)}+\left(1-I_{S}^{U}\left(x_{j}\right)\right)e^{I_{S}^{U}\left(x_{j}\right)}-1\\ +F_{S}^{U}\left(x_{j}\right)e^{\left(1-F_{S}^{U}\left(x_{j}\right)\right)}+\left(1-F_{S}^{U}\left(x_{j}\right)\right)e^{F_{S}^{U}\left(x_{j}\right)}-1 \end{array}\right]=\frac{1}{6n\left(\sqrt{e}-1\right)}\sum_{j=1}^{n}\left[\begin{array}{l} 0e^{\left(1-0\right)}+\left(1-0\right)e^{0}-1\\ +0e^{\left(1-0\right)}+\left(1-0\right)e^{0}-1\\ +e^{\left(1-1\right)}+\left(1-1\right)e-1\\ +0e^{\left(1-0\right)}+\left(1-0\right)e^{0}-1\\ +0e^{\left(1-0\right)}+\left(1-0\right)e^{0}-1\\ +e^{\left(1-0\right)}+\left(1-1\right)e-1 \end{array}\right]=0.$$

(P2) Let us consider the following function:

(3) $$f(z_{S}(x_{j}))=\frac{z_{S}(x_{j})e^{1-z_{S}(x_{j})}+(1-z_{S}(x_{j}))e^{z_{S}(x_{j})}-1}{\sqrt{e}-1}\;\mathrm{for}\;x_{j}∊X.$$

Differentiating Equation (3) with respect to *z_S_*(*x_j_*) and equating to zero, we can obtain the following results:

(4) $$\begin{array}{ll} \frac{\partial f(z_{S}(x_{j}))}{\partial z_{S}(x_{j})} & =e^{1-z_{S}(x_{j})}-z_{S}(x_{j})e^{1-z_{S}(x_{j})}-e^{z_{S}(x_{j})}+(1-z_{S}(x_{j}))e^{z_{S}(x_{j})}\\ & =(1-z_{S}(x_{j}))e^{1-z_{S}(x_{j})}-z_{S}(x_{j})e^{z_{S}(x_{j})}, \end{array}$$

(5) $$\frac{\partial f(z_{S}(x_{j}))}{\partial z_{S}(x_{j})}=(1-z_{S}(x_{j}))e^{1-z_{S}(x_{j})}-z_{S}(x_{j})e^{z_{S}(x_{j})}=0\;\mathrm{or}\;\;(1-z_{S}(x_{j}))e^{1-z_{S}(x_{j})}=z_{S}(x_{j})e^{z_{S}(x_{j})}.$$

Then, the critical point of *z_S_*(*x_j_*) is *z_S_*(*x_j_*) = 0.5 for *x_j_* ∊ *X*.

Further differentiating Equation (4) with respect to *z_S_*(*x_j_*), we get

(6) $$\frac{\partial^{2}f(z_{S}(x_{j}))}{\partial {\left(z_{S}(x_{j})\right)}^{2}}=-e^{1-z_{S}(x_{j})}-z_{S}(x_{j})e^{z_{S}(x_{j})}-e^{z_{S}(x_{j})}+(z_{S}(x_{j})-1)e^{1-z_{S}(x_{j})}.$$

Thus, we can find

$$\frac{\partial^{2}f(z_{S}(x_{j}))}{\partial {\left(z_{S}(x_{j})\right)}^{2}}<0\;\mathrm{for}\;z_{S}(x_{j})=0.5\;\mathrm{and}\;x_{j}∊X.$$

Therefore, *f*(*z_S_*(*x_j_*)) is a concave function and has the global maximum *f*(*z_S_*(*x_j_*)) = 1 at *z*_*S*_(*x_j_*) = 0.5. Thus, the SNEE of a simplified NS, *S*, can be written as the following forms:

(7) $$Y_{1}(S)=\frac{1}{3n}\sum_{j=1}^{n}\left[f\left(T_{S}\left(x_{j}\right)\right)+f\left(I_{S}\left(x_{j}\right)\right)+f\left(F_{S}\left(x_{j}\right)\right)\right]\text{ for the single}-\text{valued NS},\;S,$$

(8) $$Y_{2}(S)=\frac{1}{6n}\sum_{j=1}^{n}\left[\begin{array}{l} f\left(T_{S}^{L}\left(x_{j}\right)\right)+f\left(I_{S}^{L}\left(x_{j}\right)\right)+f\left(F_{S}^{L}\left(x_{j}\right)\right)\\ +f\left(T_{S}^{U}\left(x_{j}\right)\right)+f\left(I_{S}^{U}\left(x_{j}\right)\right)+f\left(F_{S}^{U}\left(x_{j}\right)\right) \end{array}\right]\text{ for the interval}-\text{valued NS},\;S.$$

Obviously, *Y_k_*(*S*) = 1 (*k* = 1, 2) ⇔ *S* = *A* = {<*x_j_*, [0.5, 0.5], [0.5, 0.5], [0.5, 0.5]>|*x_j_* ∊ *X*} for the interval-valued NSs, or *S* = *A* = {<*x_j_*, 0.5, 0.5, 0.5>|*x_j_* ∊ *X*} for the single-valued NSs.

(P3) Based on Equation (4), there exists the following result:

$$\frac{\partial f(z_{S}(x_{j}))}{\partial z_{S}(x_{j})}=(1-z_{S}(x_{j}))e^{1-z_{S}(x_{j})}-z_{S}(x_{j})e^{z_{S}(x_{j})}.$$

For any *z_S_*(*x_j_*) ∊ [0, 1], *f*(*z_S_*(*x_j_*)) is increasing when *z_S_*(*x_j_*) < 0.5, while *f*(*z_S_*(*x_j_*)) is decreasing when *z_S_*(*x_j_*) > 0.5.

Obviously, the closer *S* is to *A* than *P*, the fuzzier *S* is than *P*, and then *Y_k_*(*P*) ≤ *Y_k_*(*S*) (*k* = 1, 2) for *S*, *P* ∊ SNS(*X*), and *x_j_* ∊ *X*.

(P4) Since the complement of the single-valued NS, *S* = {<*x_j_*, *T_S_*(*x_j_*), *I_S_*(*x_j_*), *F_S_*(*x_j_*)>| *x_j_* ∊ *X*}, is *S^c^* = {<*x_j_*, *F_S_*(*x_j_*), 1 – *I_S_*(*x_j_*), *T_S_*(*x_j_*)>| *x_j_* ∊ *X*}, i.e., (*T_S_*(*x_j_*))^c^ = *F_S_*(*x_j_*) and (*I_S_*(*x_j_*))^c^ = 1 – *I_S_*(*x_j_*) for *x_j_* ∊ *X* and *j* = 1, 2,…, *n*, and the complement of the interval-valued NS $S=\{<x_{j},[T_{S}^{L}(x_{j}),T_{S}^{U}(x_{j})],[I_{S}^{L}(x_{j}),I_{S}^{U}(x_{j})],[F_{S}^{L}(x_{j}),F_{S}^{U}(x_{j})]>|x_{j}\in X\}$ is $S^{c}=\{<x_{j},[F_{S}^{L}(x_{j}),F_{S}^{U}(x_{j})],[1-I_{S}^{U}(x_{j}),1-I_{S}^{L}(x_{j})],[T_{S}^{L}(x_{j}),T_{S}^{U}(x_{j})]>|x_{j}\in X\}$ (i.e., ${\left[T_{S}^{L}(x_{j}),T_{S}^{U}(x_{j})\right]}^{c}=[F_{S}^{L}(x_{j}),F_{S}^{U}(x_{j})]$ and ${\left[I_{S}^{L}(x_{j}),I_{S}^{U}(x_{j})\right]}^{c}=[1-I_{S}^{U}(x_{j}),1-I_{S}^{L}(x_{j})]$ for *x_j_* ∊ *X* and *j* = 1, 2,…, *n*), then, we have *Y_k_*(*S^c^*) = *Y_k_*(*S*) (*k* = 1, 2), by using Equations (1) and (2).

This proves the theorem. ☐

It is worth noting that a single-valued NS is a special case of an interval-valued NS if there are $T_{S}^{L}(x_{j})=T_{S}^{U}(x_{j})=T_{S}^{}(x_{j})$, $I_{S}^{L}(x_{j})=I_{S}^{U}(x_{j})=I_{S}^{}(x_{j})$, and $F_{S}^{L}(x_{j})=F_{S}^{U}(x_{j})=F_{S}^{}(x_{j})$ in the interval-valued NS, $S=\{<x_{j},[T_{S}^{L}(x_{j}),T_{S}^{U}(x_{j})],[I_{S}^{L}(x_{j}),I_{S}^{U}(x_{j})],[F_{S}^{L}(x_{j}),F_{S}^{U}(x_{j})]>|x_{j}\in X\}$. In this case, Equation (2) is reduced to Equation (1).

## 4. Comparison with Other Entropy Measures for Interval-Valued NSs

Since a single-valued NS is a special case of an interval-valued NS corresponding to the equality of the two endpoints of the truth, indeterminacy, and falsity intervals in an interval-valued NS, this section only adopts an example from the literature [24] in order to compare our proposed exponential entropy measures of simplified NSs with the entropy measures of interval-valued NSs introduced by Ye and Du [24].

For convenience in our comparisons, we introduce various entropy measures of the interval-valued NS, *S*, given in previous articles [22,23,24] as follows:

(9) $$\begin{array}{l} Y_{3}(S)=1-2D_{1}(S,A)=1-\frac{1}{3n}\sum_{j=1}^{n}[\left|T_{S}^{L}(x_{j})-0.5\right|+\left|T_{S}^{U}(x_{j})-0.5\right|+\left|I_{S}^{L}(x_{j})-0.5\right|\\ \;+\left|I_{S}^{U}(x_{j})-0.5\right|+\left|F_{S}^{L}(x_{j})-0.5\right|+\left|F_{S}^{U}(x_{j})-0.5\right|], \end{array}$$

(10) $$\begin{array}{l} Y_{4}(S)=1-2D_{2}(S,A)=1-2\{\frac{1}{6n}\sum_{j=1}^{n}[{\left(T_{S}^{L}(x_{j})-0.5\right)}^{2}+{\left(T_{S}^{U}(x_{j})-0.5\right)}^{2}+{\left(I_{S}^{L}(x_{j})-0.5\right)}^{2}\\ {\;+{\left(I_{S}^{U}(x_{j})-0.5\right)}^{2}+{\left(F_{S}^{L}(x_{j})-0.5\right)}^{2}+{\left(F_{S}^{U}(x_{j})-0.5\right)}^{2}]\}}^{1/2}, \end{array}$$

(11) $$\begin{array}{l} E_{5}(S)=1-2D_{3}(S,A)=1-\frac{2}{3n}\sum_{j=1}^{n}\{\max[\left|T_{S}^{L}(x_{j})-0.5\right|,\left|T_{S}^{U}(x_{j})-0.5\right|]+\max[\left|I_{S}^{L}(x_{j})-0.5\right|,\\ \;\left|I_{S}^{U}(x_{j})-0.5\right|]+\max[\left|F_{S}^{L}(x_{j})-0.5\right|,\left|F_{S}^{U}(x_{j})-0.5\right|]\}, \end{array}$$

(12) $$\begin{array}{l} Y_{6}(S)=1-2D_{4}(S,A)=1-\frac{2}{n}\sum_{j=1}^{n}\max[\frac{1}{2}(\left|T_{S}^{L}(x_{j})-0.5\right|+\left|T_{S}^{U}(x_{j})-0.5\right|),\frac{1}{2}(\left|I_{S}^{L}(x_{j})-0.5\right|+\\ \;\left|I_{S}^{U}(x_{j})-0.5\right|),\frac{1}{2}(\left|F_{S}^{L}(x_{j})-0.5\right|+\left|F_{S}^{U}(x_{j})-0.5\right|)], \end{array}$$

(13) $$Y_{7}(S)=1-\frac{1}{2n}\sum_{j=1}^{n}\left\{[T_{S}^{L}(x_{j})+F_{S}^{L}(x_{j})]\left|I_{S}^{L}(x_{j})-{\left(I_{S}^{L}(x_{j})\right)}^{c}\right|+[T_{S}^{U}(x_{j})+F_{S}^{U}(x_{j})]\left|I_{S}^{U}(x_{j})-{\left(I_{S}^{U}(x_{j})\right)}^{c}\right|\right\},$$

(14) $$Y_{8}(S)=\frac{1}{n}\sum_{j=1}^{n}\frac{2-\left|T_{S}^{L}(x_{j})-F_{S}^{L}(x_{j})\right|-\left|T_{S}^{U}(x_{j})-F_{S}^{U}(x_{j})\right|-\left|I_{S}^{L}(x_{j})-I_{S}^{U}(x_{j})\right|}{2+\left|T_{S}^{L}(x_{j})-F_{S}^{L}(x_{j})\right|+\left|T_{S}^{U}(x_{j})-F_{S}^{U}(x_{j})\right|+\left|I_{S}^{L}(x_{j})-I_{S}^{U}(x_{j})\right|}.$$

**Example** **1.** *Let* $S=\left\{\langle x_{j},[T_{S}^{L}(x_{j}),T_{S}^{U}(x_{j})],[I_{S}^{L}(x_{j}),I_{S}^{U}(x_{j})],[F_{S}^{L}(x_{j}),F_{S}^{U}(x_{j})]\rangle |x_{j}\in X\right\}$ *be an interval-valued NS in the finite universe set X = {x_1_, x_2_, …, x_n_}. Then, we define the interval-valued NS, S^n^, for any positive real number, n, as follows:*

(15) $$S^{n}=\left\{\langle x_{j},[{\left(T_{S}^{L}(x_{j})\right)}^{n},{\left(T_{S}^{U}(x_{j})\right)}^{n}],[1-{\left(1-I_{S}^{L}(x_{j})\right)}^{n},1-{\left(1-I_{S}^{U}(x_{j})\right)}^{n}],[1-{\left(1-F_{S}^{L}(x_{j})\right)}^{n},1-{\left(1-F_{S}^{U}(x_{j})\right)}^{n}]\rangle |x_{j}\in X\right\}.$$

*When we consider the interval-valued NS, S, in the finite universe set, X = {x_1_, x_2_, x_3_, x_4_, x_5_} = {6, 7, 8, 9, 10} as S = {<6, [0.1, 0.2], [0.5, 0.5], [0.6, 0.7]>, <7, [0.3, 0.5], [0.5, 0.5], [0.4, 0.5]>, <8, [0.6, 0.7], [0.5, 0.5], [0.1, 0.2]>, <9, [0.8, 0.9], [0.5, 0.5], [0, 0.1]>, <10, [1, 1], [0.5, 0.5], [0, 0]>}, S may be viewed as “large” in X, based on the characterization of linguistic variables corresponding to these operations* [24]*: (1) S^2^ may be considered as “very large”; (2) S^3^ may be considered as “quite very large”; and (3) S^4^ may be considered as “very very large”. Thus, these operational results can be given by the interval-valued NSs, which are shown in Table 1.*

From logical consideration and human intuition, the entropy measures of these interval-valued NSs are required to satisfy the ranking order *Y_k_*(*S*) > *Y_k_*(*S*^2^) > *Y_k_*(*S*^3^) > *Y_k_*(*S*^4^) (*k* = 2–8). 

Hence, these entropy measures are calculated by Equations (2) and (9)–(14) for these cases, and all the entropy measure values are given in Table 2.

In Table 2, all entropy measure values of *Y*_2_, *Y*_3_, *Y*_4_, *Y*_5_, *Y*_6_, *Y*_8_ satisfy the required ranking order above, but the ranking order of *Y*_7_ may be unreasonable, since *Y*_7_(*S*) = 1. Obviously, the proposed exponential entropy of simplified NSs can also conform to the above required ranking order, demonstrating its rationality and effectiveness. 

## 5. Decision-Making Example Based on Entropy Measures of Interval-Valued NSs and Comparison 

In this section, the proposed exponential entropy measure of simplified NSs is applied to multiple-attribute decision-making problems. In the decision-making process, the decision maker can imply his/her provision of more useful information from the alternative if the exponential entropy value of some alternative across all attributes is smaller. Since the alternative, along with the smallest entropy value, can be considered as a significant preference and priority, we can determine the priority ranking of all alternatives, and the best one(s) corresponding to the exponential entropy values of simplified NSs (single-valued and interval-valued NSs).

For convenience in our comparisons, a decision-making example was adopted from Reference [24], where an investment company wanted to invest a sum of money into the best project. Then, the four potential alternatives were considered for the investment projects of a car company (*B*_1_), a food company (*B*_2_), a computer company (*B*_3_), and an arms company (*B*_4_). According to the set of required attributes, C = {*C*_1_, *C*_2_, *C*_3_}, where *C*_1_, *C*_2_, and *C*_3_ denote the risk, the growth, and the environmental impact, respectively, the investment company made a decision from the set of four alternatives, *B* = {*B*_1_, *B*_2_, *B*_3_, *B*_4_}. The four alternatives (projects), according to the three attributes, were only evaluated by the form of interval-valued NSs, and then all evaluation values could be constructed as the following interval-valued NS decision matrix [24]:

(16) $$D=\begin{array}{c} B_{1}\\ B_{2}\\ B_{3}\\ B_{4} \end{array}[\begin{array}{ccc} \langle C_{1},[0.4,0.5],[0.2,0.3],[0.3,0.4]\rangle & \langle C_{2},[0.4,0.6],[0.1,0.3],[0.2,0.4]\rangle & \langle C_{3},[0.7,0.9],[0.2,0.3],[0.4,0.5]\rangle \\ \langle C_{1},[0.6,0.7],[0.1,0.2],[0.2,0.3]\rangle & \langle C_{2},[0.6,0.7],[0.1,0.2],[0.2,0.3]\rangle & \langle C_{3},[0.3,0.6],[0.3,0.5],[0.8,0.9]\rangle \\ \langle C_{1},[0.3,0.6],[0.2,0.3],[0.3,0.4]\rangle & \langle C_{2},[0.5,0.6],[0.2,0.3],[0.3,0.4]\rangle & \langle C_{3},[0.4,0.5],[0.2,0.4],[0.7,0.9]\rangle \\ \langle C_{1},[0.7,0.8],[0.0,0.1],[0.1,0.2]\rangle & \langle C_{2},[0.6,0.7],[0.1,0.2],[0.1,0.3]\rangle & \langle C_{3},[0.6,0.7],[0.3,0.4],[0.8,0.9]\rangle \end{array}].$$

Then, the developed exponential entropy measure of simplified NSs was applied to the decision making problem, with information from an interval-valued NS.

Using Equation (2), we computed the exponential entropy values of interval-valued NSs for the four alternatives, which are shown in Table 3, and then we also calculated the entropy values of interval-valued NSs for the four alternatives through the entropy Equations (9)–(14) given in previous articles [22,23,24] for comparative convenience, as shown in Table 3.

In Table 3, the priority ranking order of the four alternatives based on the developed SNEE measure, *Y*_2_, was *B*_4_ ⥼ *B*_2_ ⥼ *B*_1_ ⥼ *B*_3_, corresponding to an ascending order of entropy values of *Y*_2_, where the symbol “⥼” means “superior to”. Obviously, the alternative, *B*_4_, was the best project, since the alternative with the lowest entropy value is considered as the best one. 

All priority ranking orders of the four alternatives were identical, after comparing ranking results between the developed SNEE measure and various entropy measures of interval-valued NSs given in previous articles [22,23,24] as shown in Table 3. It is clear that the decision results indicate the effectiveness of the proposed SNEE measure in the decision-making application with information from an interval-valued NS. However, this study presents the SNEE measures, including single-valued and interval-valued neutrosophic exponential entropy measures, while previous articles only gave either single-valued neutrosophic entropy measures [22] or interval-valued neutrosophic entropy measures [23,24]. Obviously, this study not only enriches the theory of simplified neutrosophic entropy, but also provides a novel way of evaluating the entropy measure of simplified neutrosophic information.

## 6. Conclusions

This paper firstly proposed the exponential entropy measures of simplified NSs, including single-valued and interval-valued neutrosophic exponential entropy measures, based on the concept of fuzzy exponential entropy. Subsequently, the properties of the exponential entropy measures for simplified NSs were discussed based on an axiomatic definition of an entropy measure taken from Reference [24], and then the proposed SNEE measure was compared with existing related entropy measures of interval-valued NSs through a numerical example, showing its effectiveness. Finally, the proposed SNEE entropy measure was applied to an actual multiple-attribute decision-making example, and then the feasibility and effectiveness of decision making in an interval-valued NS setting was demonstrated through the comparative analysis of the actual decision-making example. However, the proposed SNEE measure not only enriches the theory of simplified neutrosophic entropy, but also provides a novel way of measuring uncertain information in a simplified NS setting. In the future, we shall extend the proposed SNEE measure to propose a neutrosophic cubic exponential entropy measure, and determine its applications.

## Figures and Tables

**Table 1:** Operational results of the interval-valued neutrosophic set (NS), *S^n^*, for *n* = 1, 2, 3, 4.

*S^n^*	*x*_1_ = 6	*x*_2_ = 7	*x*_3_ = 8	*x*_4_ = 9	*x*_5_ = 10
*S*	<*x*_1_, [0.1, 0.2], [0.5, 0.5], [0.6, 0.7]>	{*x*_2_, [0.3, 0.5], [0.5, 0.5], [0.4, 0.5]>	<*x*_3_, [0.6, 0.7], [0.5, 0.5], [0.1, 0.2]>	<*x*_4_, [0.8, 0.9], [0.5, 0.5], [0, 0.1]>	<*x*_5_, [1, 1], [0.5, 0.5], [0, 0]>
*S*^2^	<6, [0.01, 0.04], [0.75, 0.75], [0.84, 0.91]>	<7, [0.09, 0.25], [0.75, 0.75], [0.64, 0.75]>	<8, [0.36, 0.49], [0.75, 0.75], [0.19, 0.36]>	<9, [0.64, 0.81], [0.75, 0.75], [0, 0.19]>	<10, [1, 1], [0.75, 0.75], [0, 0]>
*S*^3^	<6, [0.001, 0.008], [0.875, 0.875], [0.936, 0.973]>	<7, [0.027, 0.125], [0.875, 0.875], [0.784, 0.875]>	<8, [0.216, 0.343], [0.875, 0.875], [0.271, 0.488]>	<9, [0.512, 0.729], [0.875, 0.875], [0, 0.271]>	<10, [1, 1], [0.875, 0.875], [0, 0]>
*S*^4^	<6, [0.0001, 0.0016], [0.9375, 0.9375], [0.9744, 0.9919]>	<7, [0.0081, 0.0625], [0.9375, 0.9375], [0.8704, 0.9375]>	<8, [0.1296, 0.2401], [0.9375, 0.9375], [0.3439, 0.5904]>	<9, [0.4096, 0.6561], [0.9375, 0.9375], [0, 0.3439]>	<10, [1, 1], [0.9375, 0.9375], [0, 0]>

**Table 2:** Various entropy measure values of the interval-valued NSs.

*Y_k_*	*S*	*S*^2^	*S*^3^	*S*^4^
*Y*_2_	0.6954	0.5704	0.4189	0.3139
*Y*_3_ [24]	0.6067	0.3927	0.2794	0.2096
*Y*_4_ [24]	0.4450	0.3397	0.2332	0.1685
*Y*_5_ [24]	0.5467	0.3240	0.2084	0.1612
*Y*_6_ [24]	0.3000	0.2160	0.1322	0.0645
*Y*_7_ [22,24]	1.0000	0.5715	0.3581	0.2510
*Y*_8_ [23]	0.3365	0.2717	0.2662	0.2217

**Table 3:** Results and ranking orders of both the developed simplified neutrosophic exponential entropy (SNEE) measure and the existing various entropy measures of interval-valued NSs.

	*B*_1_	*B*_2_	*B*_3_	*B*_4_	Ranking Order
*Y*_2_	0.8165	0.7406	0.8429	0.3818	*B*_4_ ⥼ *B*_2_ ⥼ *B*_1_ ⥼ *B*_3_
*Y*_3_ [24]	0.6333	0.5333	0.6556	0.4444	*B*_4_ ⥼ *B*_2_ ⥼ *B*_1_ ⥼ *B*_3_
*Y*_4_ [24]	0.5654	0.4836	0.5972	0.3963	*B*_4_ ⥼ *B*_2_ ⥼ *B*_1_ ⥼ *B*_3_
*Y*_5_ [24]	0.5111	0.4222	0.5333	0.3333	*B*_4_ ⥼ *B*_2_ ⥼ *B*_1_ ⥼ *B*_3_
*Y*_6_ [24]	0.4333	0.3000	0.4667	0.2333	*B*_4_ ⥼ *B*_2_ ⥼ *B*_1_ ⥼ *B*_3_
*Y*_7_ [22,24]	0.4983	0.4933	0.5500	0.3817	*B*_4_ ⥼ *B*_2_ ⥼ *B*_1_ ⥼ *B*_3_
*Y*_8_ [23]	0.5687	0.3640	0.5728	0.3818	*B*_4_ ⥼ *B*_2_ ⥼ *B*_1_ ⥼ *B*_3_

## References

[1] Zadeh L.A. (1968). Probability measure of fuzzy events. J. Math. Anal. Appl..

[2] De Luca A.S., Termini S. (1972). A definition of nonprobabilistic entropy in the setting of fuzzy set theory. Inf. Control.

[3] Pal N.R., Pal S.K. (1989). Object background segmentation using new definitions of entropy. IEEE Proc..

[4] Verma R., Sharma B.D. (2011). On generalized exponential fuzzy entropy. Int. J. Math. Comput. Sci..

[5] Atanassov K. (1986). Intuitionistic fuzzy sets. Fuzzy Sets Syst..

[6] Atanassov K., Gargov G. (1989). Interval-valued intuitionistic fuzzy sets. Fuzzy Sets Syst..

[7] Bustince H., Burillo P. (1996). Entropy on intuitionistic fuzzy sets and on interval-valued fuzzy sets. Fuzzy Sets Syst..

[8] Szmidt E., Kacprzyk J. (2001). Entropy on intuitionistic fuzzy sets. Fuzzy Sets Syst..

[9] Valchos I.K., Sergiadis G.D. (2007). Intuitionistic fuzzy information—A pattern recognition. Pattern Recognit. Lett..

[10] Zhang Q.S., Jiang S.Y. (2008). A note on information entropy measure for vague sets. Inf. Sci..

[11] Ye J. (2010). Two effective measures of intuitionistic fuzzy entropy. Computing.

[12] Verma R., Sharma B.D. (2013). Exponential entropy on intuitionistic fuzzy sets. Kybernetika.

[13] Verma R., Sharma B.D. (2014). On intuitionistic fuzzy entropy of order-alpha. Adv. Fuzzy Syst..

[14] Verma R., Sharma B.D. (2015). R-norm entropy on intuitionistic fuzzy sets. J. Intell. Fuzzy Syst..

[15] Ye J. (2010). Multicriteria fuzzy decision-making method using entropy weights-based correlation coefficients of interval-valued intuitionistic fuzzy sets. Appl. Math. Model..

[16] Wei C.P., Wang P., Zhang Y.Z. (2011). Entropy, similarity measure of interval valued intuitionistic sets and their applications. Inf. Sci..

[17] Zhang Q.S., Xing H.Y., Liu F.C., Ye J., Tang P. (2014). Some new entropy measures for interval-valued intuitionistic fuzzy sets based on distances and their relationships with similarity and inclusion measures. Inf. Sci..

[18] Smarandache F. (1998). Neutrosophy: Neutrosophic Probability, Set, and Logic.

[19] Wang H., Smarandache F., Zhang Y.Q., Sunderraman R. (2005). Interval Neutrosophic Sets and Logic: Theory and Applications in Computing.

[20] Wang H., Smarandache F., Zhang Y.Q., Sunderraman R. (2010). Single valued neutrosophic sets. Multispace Multistruct..

[21] Ye J. (2014). A multicriteria decision-making method using aggregation operators for simplified neutrosophic sets. J. Intell. Fuzzy Syst..

[22] Majumder P., Samanta S.K. (2014). On similarity and entropy of neutrosophic sets. J. Intell. Fuzzy Syst..

[23] Aydoğdu A. (2015). On entropy and similarity measure of interval valued neutrosophic sets. Neutrosophic Sets Syst..

[24] Ye J., Du S.G. (2017). Some distances, similarity and entropy measures for interval-valued neutrosophic sets and their relationship. Int. J. Mach. Learn. Cybern..

[25] Peng J.J., Wang J.Q., Wang J., Zhang H.Y., Chen X.H. (2016). Simplified neutrosophic sets and their applications in multi-criteria group decision-making problems. Int. J. Syst. Sci..

